# Use of Foley catheter as a flap retainer

**DOI:** 10.4103/0970-0358.41119

**Published:** 2008

**Authors:** Hemant Saraiya

**Affiliations:** Sushrut Plastic Surgery Research Center and Gujarat Cancer and Research Institute, Ahmedabad, Gujarat, India

**Keywords:** Foley catheter, flap retainer

## Abstract

Keeping skin graft or a flap adherent to the underlying surface can sometimes be a difficult job, particularly inside a cavity. Different methods have been used for this function with varying success but the search is still on for an ideal pressure dressing.

## INTRODUCTION

Over the years, pressure to keep a skin graft or flap firmly opposed to the underlying wound bed has been attempted with the help of a tight dressing, tie-over dressing, elastic bandage or adherent tape. Unfortunately, the pressure may not remain effective throughout, until complete healing occurs as is necessary. We present a case where an inflated Foley′s Catheter was used to maintain gentle compression following reconstruction of an eye socket with a forehead flap.

## CASE HISTORY

**Figure 1:**
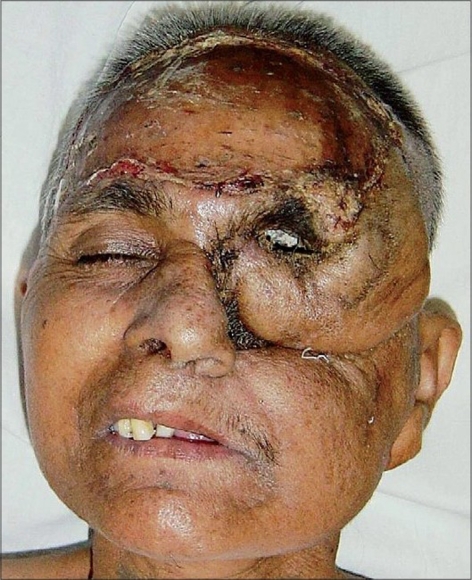
Detached forehead flap with pus discharge

**Figure 2:**
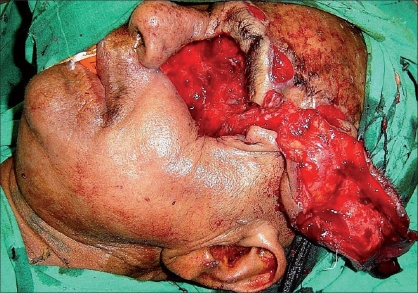
Detachment of forehead flap and removal of dirty granulation tissue

**Figure 3:**
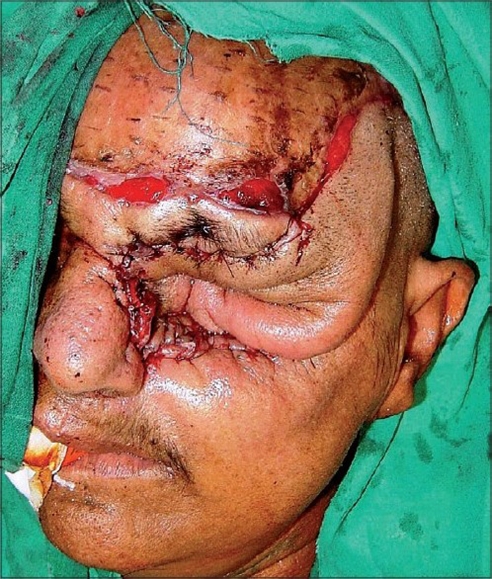
Resuturing of forehead flap to the edges

**Figure 4:**
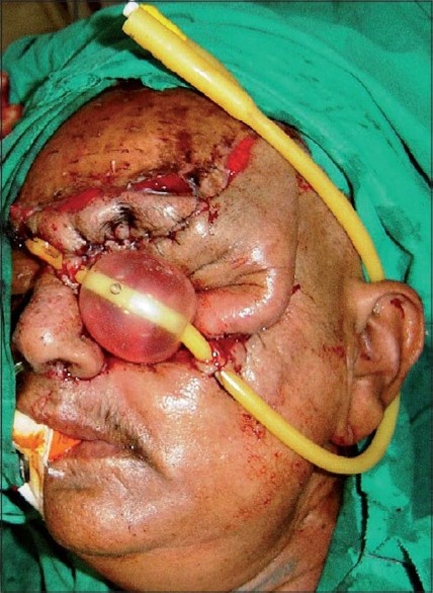
Positioning of Foley's balloon and adjustment of proper pressure

**Figure 5:**
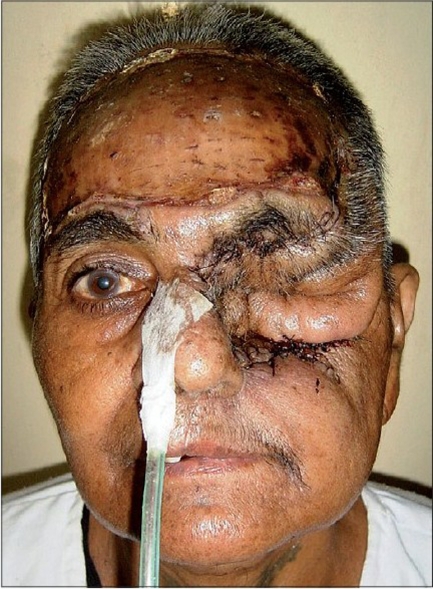
Well-adhered flap with good healing - AP view

**Figure 6:**
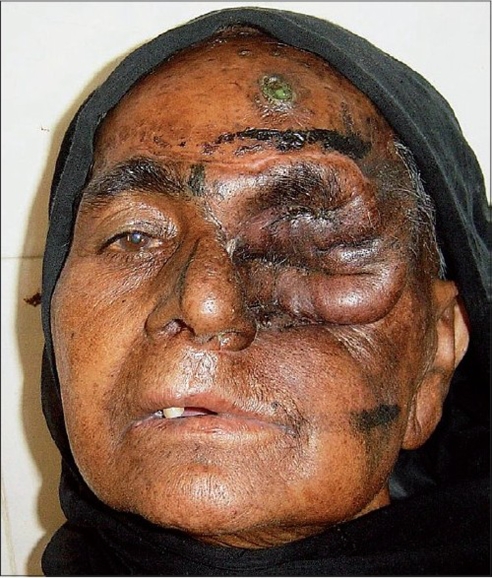
Well-adhered flap with good healing

**Figure 7:**
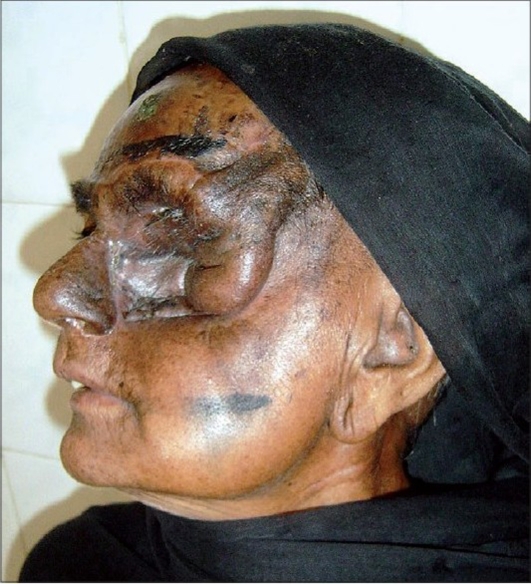
Well-adhered flap with good healing - lateral view

A 55 year-old female patient was referred to us with a raw area and discharge from the left eye socket. She had a history of swelling of the left upper alveolus and upper face for one year. She was diagnosed to have a squamous cell carcinoma of the left maxilla which had also involved the eyeball. Total left maxillectomy with left eye ball evisceration had been carried out three months earlier by the surgical oncology unit. The eye socket had been reconstructed with a split thickness skin graft. The take of the skin graft was partial and there were multiple raw areas with a purulent discharge. Repeated attempts to cover the defect with split thickness skin grafts had failed. A total forehead flap was advised by us. The flap surgery was also carried out by the surgical oncology unit itself. The patient was referred back to us after a month with a dirty foul-smelling discharge from the eye socket. On examination, the forehead flap was found to be non adherent to the socket walls [Figures 1 and 2]. There was a cavity between the flap and socket walls. After detaching the flap, thorough debridement was carried out and all infected granulations were removed [Figure 3]. The flap was reintroduced into the socket and the flap margins were sutured to the skin margins of the socket. It was felt that the flap would not remain adherent to the walls until complete healing took place unless some compression was used. To keep the flap in position, a Foley′s catheter was used. The catheter was passed through the adjoining normal skin and the bulb was positioned in such a way that the inflated bulb produced a gentle compression [Figure 4]. The compression was just enough to keep the flap in position until complete healing occurred. Radiotherapy was then started on the 15^th^ postoperative day. The follow-up showed complete coverage of the socket with a well-adhered flap [Figures 5-7].

## DISCUSSION

The catheter invented by American urologist Frederic Eugene Basil Foley is a really versatile invention.[1] Although it was intended for urinary drainage, people have found multiple uses for the catheter. Apart from its use as a drainage device, the Foley′s catheter has been used for the control of traumatic vascular injuries,[2,3] epistaxis,[4] removal of rectal and esophageal foreign bodies,[5] support for zygomatic[6] and orbital floor fractures,[7] intractable maxillary artery bleeding following complex facial fractures,[8] intra operative tissue expansion,[9,10] imperforate hymen[11] and scrotal reconstructions.[12] The use of the Foley′s catheter in our case as a compression dressing is another extension of the some.

Filling a cavity with dental material, dressing material or tie-over dressing are old methods to apply pressure, which often cannot be sustained. Also importantly, the circulation to the flap cannot be monitored. The same is true for pressure bandages and tapes. Vacuum-assisted wound closure (VAC) is also a good method but unfortunately, it is costly and not widely available.[13,14]

The above method is very effective in filling a cavity. It increases contact between the surfaces and produces effective pressure to prevent any fluid collection without jeopardizing the circulation. Pressure can be adjusted simply by adding or withdrawing fluid from the balloon. This technique is simple and cost-effective. The desired pressure can be produced on the flap and sustained so that pressure necrosis can be prevented. Needless to say, a Foley′s catheter is most easily available anywhere.
